# Network-based machine learning reveals cardiometabolic multimorbidity patterns and modifiable lifestyle factors: a community-focused analysis of NHANES 2015–2018

**DOI:** 10.1186/s12889-025-23483-9

**Published:** 2025-07-03

**Authors:** Danhui Mao, Junfang Mu, Yajing Li, Lu He, Qianhui Chai, Xin Zhao, Xiaojun Ren, Hui Cheng

**Affiliations:** 1 Third Hospital of Shanxi Medical University, Shanxi Bethune Hospital, Shanxi Academy of Medical Sciences, Tongji Shanxi Hospital, Taiyuan, Shanxi China; 2 Shanxi Medical University, Taiyuan, Shanxi China; 3 College of Computer Science and Technology, Taiyuan University of Technology, Taiyuan, Shanxi China

**Keywords:** Cardiometabolic multimorbidity, Lifestyle factors, NHANES, Community detection

## Abstract

Cardiometabolic Multimorbidity (CMM) has emerged as one of the primary threats to human health globally due to its high incidence, disability, and mortality rates. Accurate identification of CMM patterns is crucial for CMM classification and health management. However, current research on CMM pattern recognition often neglects the complex relationships among its influencing factors. Based on data from the National Health and Nutrition Examination Survey (NHANES) between 2015 and 2018, this study included 2,306 participants with an average age of 51 years, who suffered from at least two of the following conditions: hypertension, dyslipidemia, diabetes, chronic kidney disease (CKD), and hyperuricemia. By collecting demographic information, lifestyle indicators, biochemical indicators, and other characteristics of the patients, a CMM graph network was constructed with diseases as nodes and cosine similarity as the basis for calculation. The *Louvain* algorithm was used to divide the CMM graph network into communities to obtain CMM patterns. Six machine learning models (*RandomForest*, *GradientBoosting*, *SVM*, *KNN*, *Logistic Regression*, and XGBoost) were trained using these patterns as labels to identify key factors influencing CMM patterns This study identified four CMM patterns: Hypertension Predominant Group (HPG, Pattern I), Uric Acid and Dyslipidemia Coexistence Group (UADCG, Pattern II), Multiple Diseases High Group (MDHG, Pattern III), and Kidney Disease Low Group (KDLG, Pattern IV) (*Modularity* = 0.748). The distribution differences of these CMM patterns among gender, age, marital status, education level, and Family Poverty-to-Income Ratio (PIR) were statistically significant (*P* < 0.05), and so were the differences in lifestyle distribution among the four CMM patterns (*P* < 0.05). Specifically, patients in the HPG (Pattern I) pattern generally had higher nutrient intake, while those in the KDLG (Pattern IV) pattern had relatively lower intake (*P* < 0.05). Among the machine learning algorithms, *Logistic Regression* exhibited the best performance, with an *Accuracy* of 0.954 and an *AUC-ROC* area of 0.998. This study used *Louvain* and machine learning algorithm for CMM pattern detection. The features playing key roles in CMM pattern recognition included choline, iron, niacin, cholesterol, Vitamin B2 and potassium intake, which can serve as references for CMM health management.

## Introduction

Cardiometabolic Multimorbidity (CMM), with a 14.4% incidence rate, poses more severe clinical challenges than a single cardiovascular or metabolic disease, leading to increased hospitalization and mortality [1–3]. Intervention programs for different cardiovascular or metabolic diseases vary slightly, and CMM patients need to balance different intervention programs to develop a unified intervention plan. This requires an analysis of CMM patterns and epidemiological distribution characteristics across different populations [4, 5].

Most current research on comorbidity patterns and epidemiological analysis relies on cross-sectional surveys, which analyze random combinations of diseases without considering the actual combinations of diseases and the interaction relationships among influencing factors in epidemiological analysis, such as mediation, moderation, and interaction among demographics, lifestyles, and physiological characteristics, represented by complex nonlinear relationships [6–9]. Traditional statistical methods have certain limitations in revealing the complex nonlinear relationships among comorbidities [10, 11]. To effectively represent the complex nonlinear relationships among diseases in comorbidities, some scholars have described comorbidity patterns through complex networks, including network construction and feature graph representation [12]. Wang et al. used diseases as nodes and calculated the correlation strength between diseases based on the Pearson coefficient to construct a comorbidity network representing the complex nonlinear relationships among diseases. They used the *Louvain* algorithm to analyze comorbidity patterns and analyzed the characteristics of different age and gender subgroups through statistical tests [13]. However, factors influencing cardiovascular or metabolic diseases also include socioeconomic status, dietary habits, smoking, physical activity, natural aging, and other lifestyle aspects [6]. Existing research lacks adequate consideration of socioeconomic status, lifestyle, natural aging, and other factors that significantly impact diseases in the comorbidity network, and the description of epidemiological characteristics is also limited.

This paper aims to explore the complex nonlinear relationships among CMM diseases by integrating socioeconomic status, lifestyle factors, and aging biomarkers. Specifically, we seek to: identify CMM patterns; describe their epidemiological distribution across different populations; and analyze the importance of various features in CMM pattern recognition. Based on this, this study proposes to use Hypertension, Dyslipidemia, Diabetes, CKD, and Hyperuricemia as nodes, and aging-related biochemical indicators, demographic information, and lifestyle indicators as node features. A CMM graph network was constructed by calculating cosine similarity to fully represent the complex nonlinear relationships among diseases. Subsequently, the *Louvain* algorithm was used to divide the graph network into communities, divide the CMM graph network, and describe the epidemiological distribution characteristics of different CMM patterns. Finally, by comparing six machine learning algorithms: *RandomForest*, *GradientBoosting*, *SVM*, *KNN*, *Logistic Regression*, and XGBoost, the importance of various features in CMM pattern recognition was analyzed to provide insights for health management of CMM patients.

## Methods

### Patients and study design

This paper selected participants from the National Health and Nutrition Examination Survey (NHANES) conducted between 2015 and 2018 as the research subjects. The detailed design methodology of NHANES is available through the public website at https://www.cdc.gov/nchs/nhanes/. During the data preprocessing stage, we first rigorously cleaned samples with missing values to ensure the completeness and reliability of demographic information, lifestyle indicators, biochemical indicators, and disease status data. Subsequently, we conducted further screening to exclude individuals who did not have any of the five diseases (hypertension, dyslipidemia, diabetes, chronic kidney disease, and hyperuricemia) and those who had only one of these diseases. After the aforementioned screening process, a total of 2,306 individuals were included in this study, and their detailed demographic characteristics are summarized and presented in Table 2. This study has obtained formal approval from the Ethics Committee of the National Center for Health Statistics. All participants voluntarily signed informed consent forms after fully understanding the study content, ensuring ethical compliance and protecting participants'rights.

### Measurement of diseases

Hypertension was defined as the presence of either (1) an average systolic blood pressure > 140 mmHg or an average diastolic blood pressure > 90 mmHg, or (2) a doctor's diagnosis of hypertension [14]. Dyslipidemia was defined as the presence of either (1) hypercholesterolemia (total cholesterol ≥ 5.18 mmol/L), (2) hypertriglyceridemia (triglycerides ≥ 1.70 mmol/L), (3) low-density lipoprotein cholesterol (LDL-C) ≥ 3.37 mmol/L, or (4) high-density lipoprotein cholesterol (HDL-C) < 1.04 mmol/L [15]. Diabetes was defined as the presence of either (1) fasting blood glucose ≥ 7.0 mmol/L, (2) 2-h postprandial blood glucose ≥ 11.1 mmol/L, or (3) a doctor's diagnosis of diabetes [16, 17]. CKD was defined as the presence of either (1) kidney dysfunction (abnormal kidney structure or function) lasting for more than 3 months, (2) albumin-to-creatinine ratio (ACR) ≥ 30 mg/g, or (3) estimated glomerular filtration rate (eGFR) < 60 ml·min⁻^1^·(1.73 m^2^)⁻^1^ lasting for more than 3 months [18–20]. Hyperuricemia was defined as a serum uric acid level higher than 420 μmol/L (7 mg/dl) for men and higher than 357 μmol/L (6 mg/dl) for women [21]. Detailed measurement procedures are available at https://www.cdc.gov/nchs/nhanes/.

### Covariates

The variables involved in the study include demographic data, lifestyle indicators, and biochemical indicators. Demographic characteristics encompass age, gender, marital status, education level, and Family Poverty-to-Income Ratio (PIR), which is the ratio of family income to the poverty line. Lifestyle characteristics include smoking status (whether more than 100 cigarettes have been used), levels of physical activity (duration per week of Vigorous work activity, Moderate work activity, Walk or bicycle, Vigorous recreational activities, Moderate recreational activities, and Sedentary activity), as well as the average daily intake of various nutrients. Biochemical indicators relate to PhenoAge and include White blood cell count, Lymphocyte percent, Red blood cell count, Red cell distribution width, HS C-Reactive Protein, Fasting Glucose, Alkaline Phosphatase, and Hematocrit (see Tables 2 and 3 for details) [22]. Additionally, Body Mass Index (BMI) was also assessed.

### Graph network construction and CMM pattern classification

This paper systematically calculates the cosine similarity between nodes based on their feature vectors, which cover conditions such as hypertension, dyslipidemia, diabetes, CKD, and hyperuricemia. Specifically, for each node, this paper meticulously constructs a multi-dimensional feature vector that integrates multiple pieces of information, including aging-related biochemical indicators, demographic data, and lifestyle indicators. For the feature vectors of any two nodes, this paper precisely calculates their dot product by multiplying the corresponding values in each dimension and summing them up. Subsequently, the norms of these two feature vectors are calculated, which are the square roots of the sum of the squares of their values in each dimension. Finally, using the cosine similarity formula (Eq. 1), this paper derives the cosine similarity between the nodes by dividing the dot product by the product of the two norms. This process is repeated for all pairs of nodes, and in the constructed CMM network, the existence of an edge between nodes is entirely determined by the magnitude of the calculated cosine similarity values. Considerations include maximizing cosine similarity, maximizing *Modularity*, and maximizing the number of included nodes. During the preliminary experimental phase of community detection, we employed computational complexity, modularity, and robustness as the core evaluation criteria. We conducted a comparative analysis of three foundational algorithms (*Biased N-cut*, *K-means*, and *Louvain*) and their hybrid derivatives, which integrate the *Graph Attention Network* (*GAT*) specifically, *GAT* + *Biased N-cut*, *GAT* + *K-means*, and *GAT* + *Louvain*. *Louvain* algorithm was ultimately selected as the community detection method for this study. Subsequently, the CMM pattern classification are conducted based on the *Louvain* algorithm. In reality, diseases in CMM do not usually appear alone but often in combinations of multiple components. Based on this, the extended function community_multilevel of the igraph library in the *Louvain* algorithm is adopted for the detection [23, 24]. *Modularity* is used as an evaluation metric for the detection, with a higher value indicating better community division quality, i.e., nodes within communities are closely connected, while nodes between communities are sparsely connected, as shown in Eq. 2 [25]. Parameter optimization was based on the principle of maximizing *Modularity*, and a systematic Grid Search was conducted to traverse the parameter space within the ranges of *Threshold* ∈ [0.3,0.7] and *Resolution* ∈ [0.5,1.5]. Ultimately, the combination that achieved the peak *Modularity* (*Threshold* = 0.5, *Resolution* = 1) was selected. The hyperparameters for model training mainly include the similarity *Threshold* and the *Resolution* in the *Louvain* algorithm.

1 $$Similarity\left( {A,B} \right) = \;\frac{A \cdot B}{{\left\| A \right\|\left\| B \right\|}}$$

*A* and *B* are vectors, ˙ represents the dot product, and ║*A*║║*B*║ are the norms of vectors *A* and *B*, respectively.

2 $$Q = \;\frac{1}{{2{\text{m}}}}\sum\nolimits_{ij} {\left[ {A_{ij} - \frac{{k_{i} k_{j} }}{2m}} \right]} \delta \left( {c_{i} ,c_{j} } \right)$$

*A*_*ij*_ is the adjacency matrix of the graph, *k*_*i*_ and *k*_*j*_ are the degrees of nodes *i* and* j*, *m* is the total number of edges in the graph, and δ(*c*_*i*_, *c*_*j*_) is an indicator function that is 1 if nodes *i* and* j* belong to the same community and 0 otherwise.

### Machine learning model approach

Six machine learning models were employed, namely *RandomForest*, *GradientBoosting*, *SVM*, *KNN*, *Logistic Regression* and *XGBoost* [26–31]. The features used in this study within ML algorithms include demographic data, lifestyle indicators, and biochemical indicators. In this study, a comprehensive data preprocessing pipeline was implemented to ensure model robustness and interpretability. Specifically, Z-score normalization was applied to continuous variables to transform their distributions into a standard normal form with a mean of 0 and a standard deviation of 1, thereby mitigating the impact of scale differences among features. For categorical variables, one-hot encoding was employed to convert non-ordinal categorical attributes into binary vectors, enabling the model to treat each category as an independent feature without imposing ordinal relationships. Following preprocessing, the dataset was stratified into a 70% training set and a 30% test set using stratified sampling. Model performance was evaluated using *Accuracy*, *Precision*, *Recall*, *F1* score and area under the receiver operating characteristic curve (*AUC-ROC*) value. See Eqs. 3 to 6 for details. The importance ranking of the impact was analyzed based on feature weights.

3 $$Accurary = \;\frac{TP + TN}{{TP + TN + FN}}$$

4 $$Precision = \;\frac{TP}{{TP + FP}}$$

5 $${\text{Re}} call = \;\frac{TP}{{TP + FN}}$$

6 $$F1 score = \;\frac{2*(Precision\;\;*Recall)}{{Precision\;\; + Recall}}$$

True Position (TP) is the number of cases in which the classifier correctly predicts the positive case; False Position (FP) is the number of cases in which the classifier incorrectly predicts the negative case as positive; False Position (FN) is the number in which the classifier incorrectly predicts the positive case; True Position (TN) is the number in which the classifier correctly predicts the negative case as negative.

### Statistical analysis

Descriptive statistics were conducted to estimate the *Mean* (*Standard Deviation*) for continuous variables. *Frequency* (*Proportion*) was calculated for categorical variables. To determine differences in the distribution of CMM patterns with respect to characteristics, *F* test and* χ*^*2*^ test were utilized. The post-hoc multiple comparisons were conducted using the *Bonferroni* test.

**Fig. 1:**
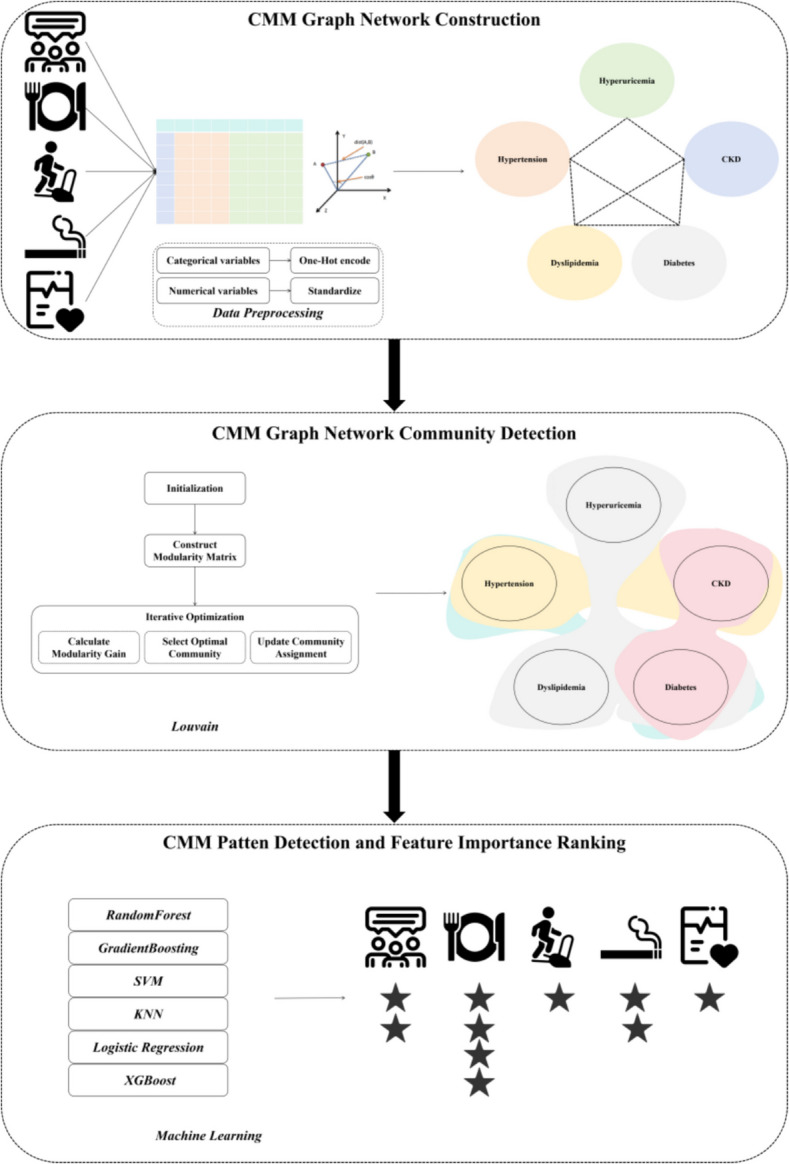
The framework of this paper

The framework of this paper was see Fig. 1. for details:

## Results

### CMM pattern analysis

Taking Hypertension, Dyslipidemia, Diabetes, CKD, and Hyperuricemia as nodes, and aging-related biochemical indicators, demographic data, and lifestyle indicators as node features, a CMM graph network is constructed by calculating cosine similarity. Considerations include maximizing cosine similarity, maximizing *Modularity*, and maximizing the number of included nodes. When *Threshold* = 0.5, based on the *Louvain* algorithm, the algorithm performance is best when *Modularity* is maximized, and through systematic search of the parameter space *Resolution* = 1, *Threshold* = 0.5 are ultimately selected, resulting in a *Modularity* of 0.748 and four CMM patterns (see Table 1).

**Table 1:** Disease in the patterns of CMM status

	CMM status *N* (*N%*)					*χ*^*2*^	*P*
Had Disease	*Pattern I* *HPG*	*Pattern II* *UADCG*	*Pattern III* *MDHG*	*Pattern IV* *KDLG*	*All*		
Hypertension	245 (52.0%)	46 (36.2%)	658 (45.0%)	88 (35.9%)	1037	21.492	＜0.001
Dyslipidemia	330 (70.1%)	88 (69.3%)	966 (66.0%)	150 (61.2%)	1534	6.337	0.096
Diabetes	150 (31.8%)	32 (25.2%)	414 (28.3%)	64 (26.1%)	660	3.952	0.267
CKD	146 (31.0%)	39 (30.7%)	446 (30.5%)	51 (20.8%)	682	10.142	0.017
Hyperuricemia	94 (20.0%)	28 (22.0%)	334 (22.8%)	34 (13.9%)	490	10.658	0.014

The results of differential tests for each disease in the four different CMM patterns are shown in Table 1. According to Table 1, Pattern III accounts for the highest proportion among the four CMM patterns (63.4%), followed by Pattern I (20.4%), and Pattern II has the lowest proportion (5.5%). For Hypertension, the highest prevalence rate is in Pattern I (52.0%), followed by Pattern III (45.0%), and the difference between groups is statistically significant (*χ*^*2*^ = 21.492, *P* < 0.001). Dyslipidemia has a high prevalence rate in all groups, but the difference between groups is not statistically significant (*P* = 0.096). The prevalence rate of Diabetes is relatively high in Pattern I (31.8%), but the overall difference between groups is not significant (*P* = 0.267). The prevalence rates of CKD in Pattern I and Pattern III are similar, but it is significantly reduced in Pattern IV (20.8%), and the difference between groups is statistically significant (*χ*^*2*^ = 10.142, *P* = 0.017). Hyperuricemia has higher prevalence rates in Pattern II and Pattern III, at 22.0% and 22.8% respectively, and is lowest in Pattern IV (13.9%), with the difference between groups also being statistically significant (*χ*^*2*^ = 10.658, *P* = 0.014). Based on the above disease distribution characteristics, Pattern I is named the Hypertension Predominant Group (HPG). Pattern II is named the Uric Acid and Dyslipidemia Coexistence Group (UADCG). Pattern III has high prevalence rates of multiple diseases, such as Hypertension (45.0%) and Dyslipidemia (66.0%), showing characteristics of multiple diseases coexistence, hence it is named the Multiple Diseases High Group (MDHG). The prevalence rate of CKD in Pattern IV is the lowest (20.8%), and the prevalence rate of Hyperuricemia is also relatively low (13.9%), so it is named the Kidney Disease Low Group (KDLG).

### Distribution characteristics of different CMM patterns in populations

The distribution characteristics of the four CMM patterns across different populations are detailed in Table 2. According to Table 2, the differences in the distribution of the four CMM patterns among different genders, ages, marital statuses, education levels, and Family PIR are statistically significant (*P* < 0.001). In terms of gender, the proportion of males and females is unevenly distributed across patterns (*χ*^*2*^ = 134.041, *P* < 0.001). Specifically, in the HPG (Pattern I) pattern, females account for 40.6% and males for 59.4%, while the proportions of males are relatively higher in the Pattern II (UADCG) and Pattern IV (KDLG) patterns, especially inPattern IV (KDLG), where males account for 74.3%. Regarding marital status, the differences between patterns are also statistically significant (*χ*^*2*^ = 59.643, *P* < 0.001), with the highest proportion of married individuals in the HPG (Pattern I) pattern (63.1%), while the proportions of unmarried individuals or those cohabiting with a partner are relatively higher in other patterns. In terms of education level, the differences between patterns are equally significant (*χ*^*2*^ = 54.719, *P* < 0.001). In the HPG (Pattern I) pattern, there is a higher proportion of individuals with higher education levels, specifically 31.45% with college degrees or above, which is more than in other patterns. Conversely, lower education levels are more prevalent in Pattern II (UADCG) and Pattern III (MDHG), especially in Pattern II (UADCG), where individuals with 9–11 years of education account for 13.4%. Bonferroni multiple comparison results indicate that the age of the HPG (Pattern I) pattern is significantly higher than that of the other patterns, and the age of the Pattern IV (KDLG) pattern is significantly lower than that of the MDHG (Pattern III) pattern (*P* < 0.05). The Family PIR of the HPG (Pattern I) pattern is significantly higher than that of the other patterns (*P* < 0.05). The BMI of the HPG (Pattern I) pattern is significantly higher than that of the MDHG (Pattern III) and Pattern IV (KDLG) patterns (*P* < 0.05). Additionally, this pattern has a lower white blood cell count and a higher lymphocyte percent, both of which are significantly different from the MDHG (Pattern III) and Pattern IV (KDLG) patterns (*P* < 0.05). The UADCG (Pattern II) pattern has the highest lymphocyte percent, and its red blood cell count is also significantly higher than that of the MDHG (Pattern III) and KDLG (Pattern IV) patterns (*P* < 0.05). Furthermore, the HPG (Pattern I) pattern has lower red cell distribution width and alkaline phosphatase levels, which are significantly lower than those of the MDHG (Pattern III) pattern (*P* < 0.05). Hematocrit is highest in the HPG (Pattern I) pattern, significantly higher than in the other patterns (*P* < 0.05).

**Table 2:** Demographic characteristics of the subjects in different CMM status

	CMM status *Mean (SD*) *OR N* (*N%*)					*F/χ*^*2*^	*P*
	*Pattern I* *HPG*	*Pattern II* *UADCG*	*Pattern III* *MDHG*	*Pattern IV* *KDLG*	*All*		
Age^a^	53.909 (16.472)	48.307 (17.066)	50.758 (17.781)	46.192 (17.050)	50.781 (17.525)	8.369	＜0.001
Gender^b^						134.041	＜0.001
Females	191 (40.6%)	48 (37.8%)	870 (59.5%)	63 (25.7%)	1172		
Males	280 (59.4%)	79 (62.2%)	593 (40.5%)	182 (74.3%)	1134		
Marital status^b^						59.643	＜0.001
Married	297 (63.1%)	71 (55.9%)	714 (48.8%)	119 (48.6%)	1201		
Widowed	35 (7.4%)	5 (3.9%)	110 (7.5%)	6 (2.4%)	156		
Divorced	41 (8.7%)	11 (8.7%)	170 (11.6%)	32 (13.1%)	254		
Separated	12 (2.5%)	9 (7.1%)	61 (4.2%)	4 (1.6%)	86		
Never married	60 (12.7%)	18 (14.2%)	254 (17.4%)	49 (20.0%)	381		
Living with partner	26 (5.5%)	13 (10.2%)	154 (10.5%)	35 (14.3%)	228		
Education level^b^						54.719	＜0.001
Less than 9th grade	27 (5.7%)	9 (7.1%)	126 (8.6%)	12 (4.9%)	174		
9-11th grade	44 (9.3%)	17 (13.4%)	166 (11.3%)	39 (15.9%)	266		
High school graduate/GED or equivalent	81 (17.2%)	30 (23.6%)	376 (25.7%)	60 (24.5%)	547		
Some college or AA degree	171 (36.3%)	56 (44.1%)	429 (29.3%)	84 (34.3%)	740		
College graduate or above	148 (31.5%)	15 (11.8%)	366 (25.0%)	50 (20.4%)	579		
Family PIR^a^	3.156 (1.594)	2.399 (1.531)	2.391 (1.579)	2.196 (1.486)	2.527 (1.602)	32.764	＜0.001
BMI^a^	31.044 (7.841)	29.598 (6.802)	29.130 (7.107)	29.312 (7.189)	29.566 (7.290)	8.369	＜0.001
White blood cell count^a^	6.473 (1.764)	6.771 (1.964)	6.841 (2.018)	7.209 (2.188)	6.801 (1.994)	7.958	＜0.001
Lymphocyte percent^a^	33.292 (8.656)	35.725 (9.906)	31.323 (8.787)	28.535 (8.225)	31.672 (8.907)	25.629	＜0.001
Red blood cell count^a^	4.840 (0.497)	4.936 (0.530)	4.790 (0.523)	4.738 (0.548)	4.803 (0.522)	5.127	0.002
Red cell distribution width^a^	13.621 (1.127)	13.791 (1.216)	13.855 (1.414)	13.938 (1.540)	13.813 (1.367)	4.276	0.005
HS C-Reactive Protein^a^	3.354 (5.812)	2.934 (4.382)	4.120 (8.717)	4.253 (6.877)	3.912 (7.829)	1.959	0.118
Fasting Glucose^a^	111.017 (39.077)	112.968 (47.280)	111.896 (36.553)	109.282 (31.795)	111.498 (37.267)	0.436	0.727
Alkaline Phosphatase^a^	79.554 (35.667)	78.850 (38.296)	87.532 (55.262)	83.037 (44.595)	84.947 (49.997)	3.894	0.009
Hematocrit^a^	42.525 (3.682)	41.200 (4.651)	41.416 (4.154)	41.531 (3.887)	41.643 (4.086)	9.480	＜0.001

^a^The description of these variables were *Mean* (*SD*)

^b^The description of these variables were *N* (*N%*)

The distribution characteristics of the four CMM patterns in lifestyle factors are detailed in Table 3. According to Table 3, the differences in the distribution of the four CMM patterns across lifestyle factors, except for walk or bicycle physical activity, are statistically significant (*P* < 0.001). Among them, the UADCG (Pattern II) pattern has the highest proportion of individuals who have smoked more than 100 cigarettes, reaching 57.5% (*χ*^*2*^ = 18.595, *P* < 0.001). Bonferroni multiple comparison results show that the UADCG (Pattern II) pattern has significantly higher physical activity levels than the other patterns (*P* < 0.05). The HPG (Pattern I) pattern has significantly higher intakes of protein, carbohydrates, dietary fiber, and various vitamins and minerals (such as vitamin E, vitamin C, vitamin D, calcium, magnesium, iron, zinc, copper, etc.) than the MDHG (Pattern III) pattern and the UADCG (Pattern II) pattern (*P* < 0.05). Additionally, the cholesterol intake of the HPG (Pattern I) pattern is significantly higher than that of the UADCG (Pattern II) and MDHG (Pattern III) patterns (*P* < 0.05). Furthermore, the UADCG (Pattern II) pattern also shows higher intakes of various nutrients (such as vitamin A, vitamin B1, vitamin B2, vitamin B6, folic acid, choline, vitamin B12, etc.), although there is still a gap compared to the HPG (Pattern I) pattern. Notably, the KDLG (Pattern IV) pattern has relatively higher intakes of various nutrients, especially vitamin A, phosphorus, and calcium, which are significantly higher than in other patterns (*P* < 0.05).

**Table 3:** Lifestyle characteristics of the subjects in different CMM status

	CMM status *Mean (SD*) *OR N* (*N%*)					*F/χ*^*2*^	*P*
	*Pattern I* *HPG*	*Pattern II* *UADCG*	*PatternIII* *MDHG*	*Pattern IV* *KDLG*	*All*		
Smoking status^b^ Smoked at least 100 cigarettes in life	218 (46.3%)	73 (57.5%)	598 (40.9%)	120 (49.0%)	1009	18.595	<0.001
Vigorous work activity^a^	157.113 (410.032)	1616.921 (1397.569)	97.946 (302.866)	374.571 (763.235)	223.076 (617.419)	353.85	<0.001
Moderate work activity^a^	399.781 (2358.913)	1164.685 (1074.212)	282.013 (583.865)	469.306 (799.639)	374.578 (1233.907)	21.191	<0.001
Walk or bicycle^a^	55.881 (214.567)	107.559 (296.766)	64.515 (273.906)	92.612 (393.905)	68.108 (280.002)	1.848	0.136
Vigorous recreational activities^a^	63.457 (143.852)	154.724 (388.062)	41.636 (116.055)	80.229 (148.127)	56.422 (155.108)	24.403	<0.001
Moderate recreational activities^a^	91.306 (151.082)	194.803 (379.348)	72.826 (170.063)	123.674 (251.381)	88.721 (196.127)	18.627	<0.001
Sedentary activity^a^	401.391 (491.188)	266.378 (181.333)	342.239 (200.361)	340.653 (182.587)	349.974 (284.529)	9.329	<0.001
Protein^a^	100.721 (25.212)	61.721 (26.146)	63.382 (22.168)	130.382 (45.261)	78.036 (35.254)	603.883	<0.001
Carbohydrate^a^	275.29 (88.015)	201.988 (101.539)	209.545 (81.757)	375.904 (151.098)	240.232 (108.099)	251.655	<0.001
Dietary fiber^a^	20.998 (9.225)	13.416 (8.946)	14.016 (7.283)	26.205 (13.74)	16.704 (9.69)	188.814	<0.001
Saturated fatty acids^a^	36.03 (13.238)	20.232 (10.038)	20.822 (9.542)	42.885 (19.642)	26.24 (14.474)	382.232	<0.001
Monounsaturated fatty acids^a^	39.623 (13.804)	21.828 (11.603)	22.464 (9.38)	44.745 (19.276)	28.301 (14.84)	428.123	<0.001
Polyunsaturated fatty acids^a^	26.192 (11.084)	14.012 (7.952)	15.645 (7.534)	29.443 (14.586)	19.175 (10.848)	270.186	<0.001
Cholesterol^a^	467.902 (200.961)	213.732 (159.362)	228.522 (127.902)	488.849 (308.896)	304.259 (208.052)	336.152	<0.001
Vitamin E^a^	11.729 (5.621)	5.946 (3.221)	6.789 (3.534)	14.052 (7.454)	8.523 (5.333)	281.068	<0.001
Retinol^a^	475.121 (231.611)	221.047 (166.408)	292.743 (203.996)	827.102 (603.779)	382.818 (326.726)	290.468	<0.001
Vitamin A^a^	721.091 (371.743)	364.181 (263.716)	472.139 (349.309)	1107.604 (673.942)	584.557 (448.371)	212.41	<0.001
Vitamin B1^a^	1.764 (0.547)	1.186 (0.56)	1.291 (0.542)	2.807 (1.07)	1.543 (0.783)	451.883	<0.001
Vitamin B2^a^	2.426 (0.746)	1.717 (1.09)	1.531 (0.631)	3.588 (1.415)	1.943 (1.044)	532.223	<0.001
Niacin^a^	28.15 (9.267)	21.106 (12.314)	19.99 (8.35)	45.233 (17.739)	24.4 (12.856)	460.011	<0.001
Vitamin B6^a^	2.279 (0.735)	1.711 (1.404)	1.569 (0.769)	4.013 (2.27)	1.981 (1.307)	383.314	<0.001
Folate^a^	421.431 (143.556)	268.898 (131.559)	311.854 (146.824)	707.551 (321.959)	373.91 (212.461)	395.205	<0.001
Choline^a^	466.792 (131.847)	250.007 (123.028)	252.747 (99.883)	532.872 (221.019)	326.076 (167.912)	595.816	<0.001
Vitamin B12^a^	5.092 (2.564)	3.636 (3.419)	3.214 (2.158)	10.362 (9.103)	4.38 (4.302)	268.796	<0.001
Vitamin C^a^	97.096 (77.475)	67.35 (75.682)	64.189 (58.09)	123.535 (111.315)	77.39 (73.558)	64.882	<0.001
Vitamin D^a^	5.266 (4.179)	2.865 (2.88)	3.142 (2.792)	10.206 (8.11)	4.311 (4.559)	234.366	<0.001
Vitamin K^a^	149.644 (134.98)	83.16 (96.272)	102.4 (120.535)	155.814 (151.289)	116.665 (128.202)	27.876	<0.001
Calcium^a^	1074.03 (382.539)	683.284 (327.87)	732.256 (340.822)	1572.6 (620.635)	888.648 (474.087)	381.155	<0.001
Phosphorus^a^	1691.055 (390.797)	1022.347 (392.88)	1056.046 (355.769)	2227.816 (711.208)	1308.385 (581.466)	728.813	<0.001
Magnesium^a^	367.259 (110.817)	246.764 (107.479)	236.857 (87.308)	467.706 (172.313)	288.563 (132.602)	446.708	<0.001
Iron^a^	15.601 (4.505)	10.243 (5.386)	11.277 (4.978)	25.696 (10.212)	13.635 (7.273)	482.263	<0.001
Zinc^a^	13.008 (3.811)	8.291 (5.408)	8.259 (3.438)	20.093 (16.979)	10.488 (7.556)	261.751	<0.001
Copper^a^	1.484 (0.546)	0.968 (0.552)	0.941 (0.367)	1.908 (1.439)	1.156 (0.704)	227.41	<0.001
Sodium^a^	4215.527 (1293.447)	2632.803 (1151.671)	2801.813 (1044.642)	5269.396 (1953.755)	3343.423 (1507.836)	388.116	<0.001
Potassium^a^	3243.682 (825.286)	2203.102 (941.963)	2070.932 (733.943)	4012.51 (1302.872)	2524.027 (1089.053)	516.762	<0.001
Selenium^a^	142.409 (44.964)	86.656 (41.239)	90.351 (35.262)	187.164 (81.941)	111.066 (55.911)	429.263	<0.001
Caffeine^a^	182.076 (188.95)	251.126 (458.15)	116.212 (138.925)	153.192 (156.449)	141.024 (187.097)	32.414	<0.001
Theobromine^a^	55.2 (84.219)	29.85 (63.391)	25.052 (41.983)	42.082 (68.169)	33.283 (58.531)	35.144	<0.001
Alcohol^a^	12.491 (32.238)	6.909 (15.277)	6.408 (18.449)	9.766 (22.986)	8.035 (22.419)	9.468	<0.001

^a^The description of these variables were *Mean* (*SD*)

^b^The description of these variables were *N* (*N%*)

### Machine learning-based detection of CMM patterns

**Fig. 2:**
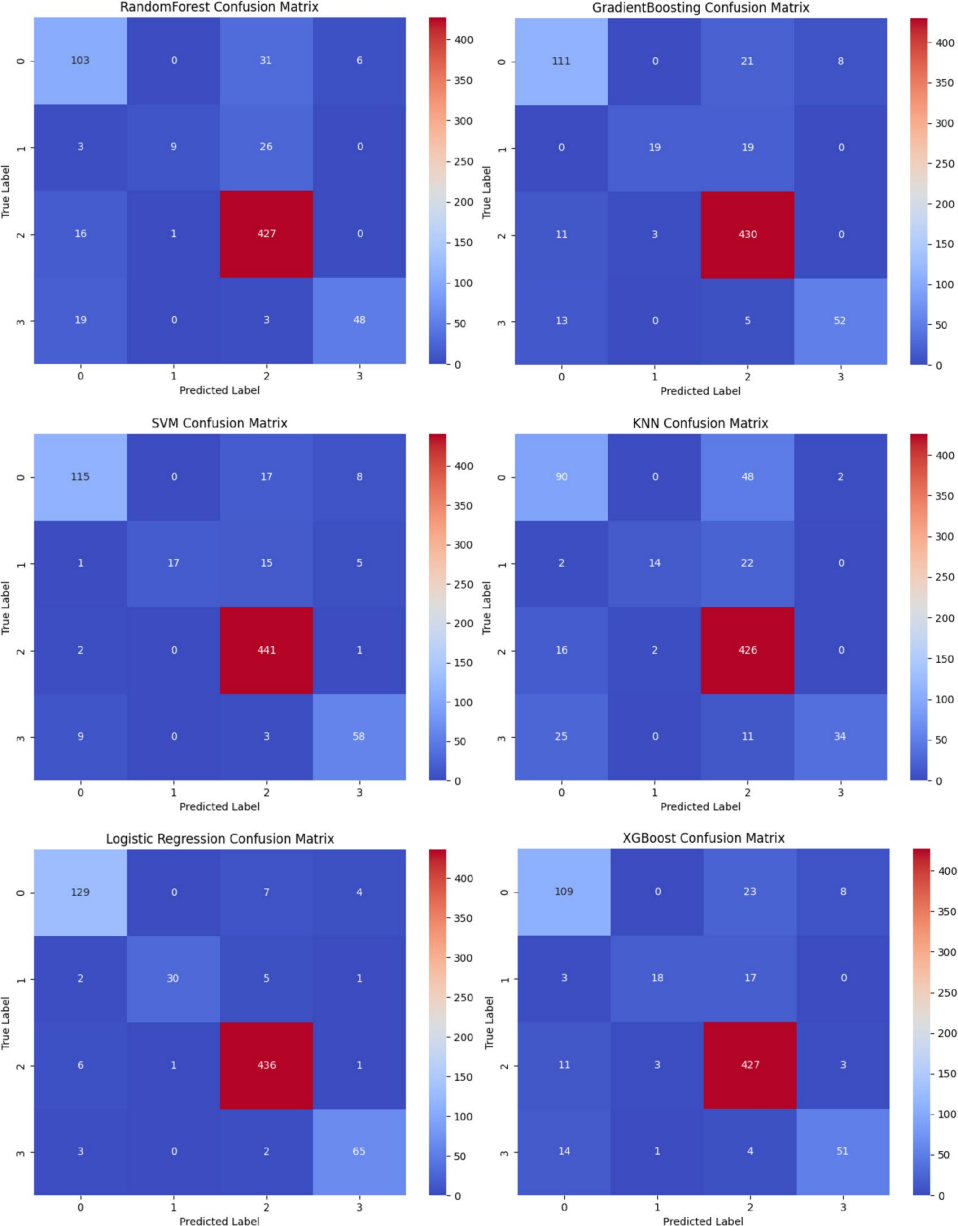
Local confusion matrix for each model

The recognition performance of six different types of machine learning algorithms in predicting CMM patterns is detailed in Table 4. According to Table 4, compared to the other five algorithms, *Logistic Regression* achieves the highest *Accuracy* (0.954), *Precision* (0.954), *Recall* (0.953), *F1* score (0.953), and an area under the *AUC-ROC* curve of 0.998. The confusion matrices of each algorithm are shown in Fig. 2. Figure 2. indicates that *Logistic Regression* has the highest performance. Considering various evaluation indicators of the algorithms comprehensively, the optimal machine learning algorithm for CMM pattern recognition in this study is *Logistic Regression*.

**Table 4:** Fit indices for machine learning models

Models	*Accuracy*	*Precision*	*Recall*	*F1 Score*	*AUC-ROC*
*RandomForest*	0.841	0.836	0.817	0.826	0.964
*GradientBoosting*	0.884	0.882	0.878	0.880	0.972
*SVM*	0.912	0.914	0.900	0.906	0.990
*KNN*	0.815	0.820	0.785	0.802	0.898
*Logistic Regression*	0.954	0.954	0.953	0.953	0.998
*XGBoost*	0.874	0.871	0.867	0.869	0.973

### Feature importance analysis in CMM pattern detection

**Fig. 3:**
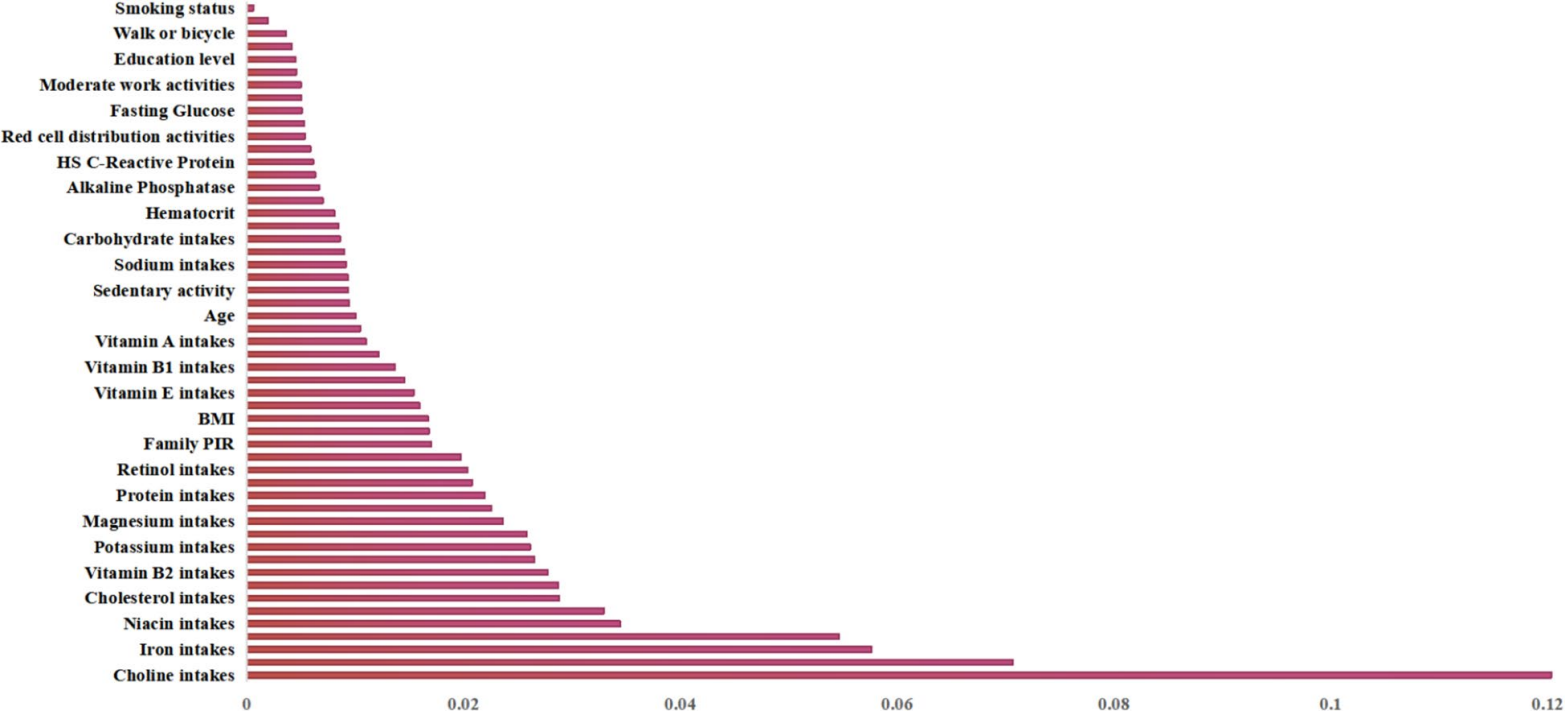
The importance of different features for CMM pattern detection

Considering various evaluation indicators of the algorithms comprehensively, *GradientBoosting* is the optimal choice among *RandomForest*, *GradientBoosting*, and XGBoost. Feature importance in CMM pattern recognition is analyzed using the *GradientBoosting* algorithm, as shown in Fig. 3. According to Fig. 3, the features that play important roles in CMM pattern recognition include choline intakes, iron intakes, niacin intakes, cholesterol intakes, vitamin B2 intakes, and potassium intakes. In contrast, features that play lesser roles include smoking status, walk or bicycle, and education level.

### Differential impact of lifestyle factors on CMM patterns

Since the Pattern II UADCG group had relatively low levels of almost all diseases, it was selected as the reference group for comparison. Since the Pattern II UADCG group had relatively low levels of almost all diseases, it was selected as the reference group for comparison. From Tables 5, 6 and 7 it is showed that patients with higher choline intake were 3.6% more likely of being in Pattern I (*OR* = 1.036, *95% CI*: 1.01–1.063, *P* = 0.006) and 4.9% more likely of being in Pattern IV (*OR* = 1.049, *95% CI*: 1.011–1.088, *P* = 0.011). Patients with higher iron intake were 138.8% more likely of being in Pattern IV (*OR* = 2.388, 95% *CI*: 1.497–3.81,* P* < 0.001). And patients with higher cholesterol intake were 19% more likely of being in Pattern I (*OR* = 1.019, *95% CI*: 1.003–1.035, *P* = 0.017), while patients with higher potassium intake were 2% less likely of being in Pattern III (*OR* = 0.998, *95% CI:* 0.995–1.000, *P* = 0.038). See Table 5 for more details (Table 5).

**Table 5:** Physical activity factors impact on CMM patterns

	*Pattern I* *HPG~*		*Pattern III* *MDHG~*		*Pattern IV* * KDLG~*	
	*Odds Ratio*	*P*	*Odds Ratio*	*P*	*Odds Ratio*	*P*
	*(95% Confidence Interval)*		*(95% Confidence Interval)*		*(95% Confidence Interval)*	
Vigorous work activity	0.986(0.983,0.989)	＜0.001	0.988(0.985,0.991)	＜0.001	0.987(0.984,0.991)	＜0.001
Moderate recreational activities	1(0.999,1.001)	0.996	1(0.999,1.001)	0.871	0.999(0.997,1.001)	0.256
Walk or bicycle	1.004(1,1.008)	0.080	1.004(1,1.008)	0.078	1(0.995,1.005)	0.949
Vigorous recreational activities	0.994(0.988,0.999)	0.018	0.985(0.98,0.99)	＜0.001	0.976(0.967,0.985)	＜0.001
Moderate work activities	0.993(0.988,0.997)	0.001	0.993(0.99,0.996)	＜0.001	0.994(0.989,1)	0.059
Sedentary activity	1.008(1.004,1.012)	＜0.001	0.999(0.996,1.003)	0.766	1.006(0.999,1.013)	0.108

^~^The Pattern II UADCG was the reference

**Table 6:** Dietary factors impact on CMM patterns

	*Pattern IHPG~*		*Pattern IIIMDHG~*		*Pattern IV KDLG~*	
	*Odds Ratio*	*P*	*Odds Ratio*	*P*	*Odds Ratio*	*P*
	*(95% Confidence Interval)*		*(95% Confidence Interval)*		*(95% Confidence Interval)*	
Protein intakes	1.037(0.926,1.162)	0.530	1.016(0.919,1.123)	0.758	1.025(0.887,1.185)	0.740
Carbohydrate intakes	0.964(0.927,1.003)	0.073	0.969(0.935,1.004)	0.085	0.994(0.946,1.044)	0.803
Sugars intake	1.01(0.963,1.06)	0.682	1.06(1.014,1.108)	0.009	1.012(0.953,1.076)	0.694
Dietary fiber intakes	1.244(0.974,1.59)	0.081	0.981(0.786,1.224)	0.863	1.043(0.772,1.409)	0.785
Saturated fatty acids intakes	1.252(1.064,1.474)	0.007	1.015(0.887,1.161)	0.830	1.164(0.907,1.494)	0.232
Monounsaturated fatty acids intakes	1.414(1.122,1.782)	0.003	1.129(0.916,1.39)	0.255	1.184(0.855,1.639)	0.309
Polyunsaturated fatty acids intakes	1.047(0.81,1.352)	0.728	1.046(0.823,1.33)	0.714	1.016(0.73,1.413)	0.925
Cholesterol intakes	1.019(1.003,1.035)	0.017	0.993(0.979,1.008)	0.353	1.003(0.981,1.025)	0.801
Vitamin E intakes	1.709(1.095,2.668)	0.018	1.344(0.883,2.047)	0.168	2.175(1.31,3.613)	0.003
Retinol intakes	1.009(0.998,1.019)	0.099	1.01(1.001,1.02)	0.036	1.022(1.01,1.034)	＜0.001
Vitamin A intakes	1.004(0.998,1.01)	0.156	1.004(0.998,1.009)	0.174	1.006(0.999,1.013)	0.072
Vitamin B1 intakes	0.5(0.028,8.994)	0.638	0.704(0.048,10.41)	0.798	99.696(2.277,4365.136)	0.017
Vitamin B2 intakes	1.269(0.044,36.854)	0.890	0.234(0.011,5.01)	0.353	10.593(0.149,751.005)	0.278
Niacin intakes	0.906(0.68,1.207)	0.499	1.088(0.841,1.409)	0.520	1.182(0.796,1.756)	0.406
Vitamin B6 intakes	0.729(0.085,6.21)	0.772	1.48(0.275,7.957)	0.648	321.117(13.041,7907.264)	＜0.001
Folate intakes	1.007(0.993,1.021)	0.330	1.011(0.997,1.024)	0.122	1.026(1.009,1.042)	0.002
Choline intakes	1.036(1.01,1.063)	0.006	0.998(0.976,1.02)	0.857	1.049(1.011,1.088)	0.011
Vitamin B12 intakes	0.706(0.437,1.141)	0.155	0.623(0.414,0.938)	0.024	1.497(0.753,2.975)	0.250
Vitamin C intakes	1.01(0.993,1.027)	0.263	0.985(0.97,0.999)	0.041	1.016(0.994,1.039)	0.154
Vitamin D intakes	1.126(0.776,1.634)	0.531	0.784(0.564,1.09)	0.147	2.349(1.437,3.838)	0.001
Vitamin K intakes	0.987(0.976,0.998)	0.017	1(0.991,1.01)	0.959	0.981(0.967,0.996)	0.011
Calcium intakes	1.004(0.999,1.01)	0.139	1.005(1,1.01)	0.069	1.009(1.002,1.016)	0.008
Phosphorus intakes	1.004(0.998,1.01)	0.186	1(0.994,1.005)	0.965	1.004(0.994,1.014)	0.420
Magnesium intakes	1.001(0.976,1.026)	0.945	0.992(0.969,1.015)	0.479	0.997(0.967,1.028)	0.851
Iron intakes	1.254(0.867,1.814)	0.228	1.239(0.884,1.737)	0.214	2.388(1.497,3.81)	＜0.001
Zinc intakes	0.873(0.599,1.272)	0.480	0.922(0.678,1.254)	0.603	1.861(1.114,3.108)	0.018
Copper intakes	9.703(0.163,578.467)	0.276	0.544(0.012,24.028)	0.753	17.907(0.096,3325.404)	0.279
Sodium intakes	1(0.999,1.002)	0.750	1(0.999,1.002)	0.601	1(0.998,1.002)	0.668
Potassium intakes	0.999(0.997,1.002)	0.642	0.998(0.995,1)	0.038	0.999(0.995,1.003)	0.659
Selenium intakes	1.067(1.01,1.128)	0.021	1.058(1.005,1.114)	0.031	1.064(0.994,1.138)	0.073
Caffeine intakes	1.003(0.997,1.01)	0.315	0.998(0.992,1.004)	0.491	0.988(0.978,0.998)	0.016
Heobromine intake	1.046(1.018,1.074)	0.001	0.991(0.968,1.015)	0.468	0.986(0.956,1.018)	0.397
Alcohol intake	1.122(1.045,1.205)	0.002	1.048(0.983,1.117)	0.151	0.993(0.893,1.105)	0.903

^~^The Pattern II UADCG was the reference

**Table 7:** Other factors impact on CMM patterns

	*Pattern IHPG~*		*Pattern IIIMDHG~*		*Pattern IV KDLG~*	
	*Odds Ratio*	*P*	*Odds Ratio*	*P*	*Odds Ratio*	*P*
	*(95% Confidence Interval)*		*(95% Confidence Interval)*		*(95% Confidence Interval)*	
Family PIR	1.818(0.93,3.555)	0.081	0.563(0.311,1.02)	0.058	0.158(0.05,0.5)	0.002
BMI	1.639(1.405,1.911)	＜0.001	0.962(0.87,1.063)	0.444	1.36(1.142,1.619)	0.001
White blood cell count	0.347(0.213,0.565)	＜0.001	0.446(0.301,0.662)	＜0.001	4.093(1.742,9.614)	0.001
Lymphocyte percent	0.68(0.595,0.777)	＜0.001	0.625(0.547,0.713)	＜0.001	0.356(0.279,0.454)	＜0.001
Red blood cell count	3.909(0.684,22.323)	0.125	0.111(0.025,0.485)	0.003	0.054(0.005,0.62)	0.019
Red cell distribution activities	1.018(0.492,2.106)	0.961	1.858(0.953,3.62)	0.069	3.984(1.629,9.741)	0.002
HS C-Reactive Protein	0.883(0.81,0.962)	0.005	0.953(0.9,1.01)	0.102	1.049(0.855,1.288)	0.645
Fasting Glucose	0.993(0.967,1.021)	0.634	0.989(0.964,1.015)	0.391	0.981(0.943,1.02)	0.330
Alkaline Phosphatase	0.969(0.949,0.99)	0.004	1.02(1.002,1.037)	0.025	1.004(0.979,1.029)	0.760
Hematocrit	2.697(2.038,3.568)	＜0.001	1.172(0.971,1.413)	0.098	0.875(0.612,1.25)	0.463
Age	1.041(0.971,1.115)	0.259	0.906(0.853,0.962)	0.001	0.934(0.85,1.026)	0.153
Gender^a^						
Femal	11.428(1.518,86.056)	0.018	0.217(0.038,1.228)	0.084	29.284(1.127,760.927)	0.042
Education level^a^						
Less than 9th grade	0.335(0.009,12.059)	0.549	0.532(0.023,12.229)	0.693	69.855(0.085,57261.558)	0.215
9-11th grade	0.108(0.005,2.501)	0.165	0.129(0.011,1.497)	0.101	215.106(0.968,47819.841)	0.051
High school graduate/GED or equivalent	1.513(0.129,17.725)	0.741	3.717(0.428,32.307)	0.234	84.843(0.616,11685.6)	0.077
Some college or AA degree	7.979(0.779,81.717)	0.080	0.483(0.063,3.712)	0.484	289.62(5.492,15272.958)	0.005
Marital status^a^						
Married	5.219(0.196,139.228)	0.324	0.322(0.022,4.809)	0.412	0.027(0,1.775)	0.091
Widowed	8.976(0.086,935.365)	0.355	2.083(0.031,140.701)	0.733	0.079(0,84.968)	0.476
Divorced	0.732(0.02,26.569)	0.865	0.381(0.02,7.397)	0.524	0.36(0.002,77.63)	0.709
Separated	29.732(0.175,5043.49)	0.195	0.491(0.006,37.539)	0.748	0(0,24194325184.62)	0.564
Never married	6.543(0.144,296.306)	0.334	2.713(0.105,70.321)	0.548	0.645(0.003,132.024)	0.872
Smoking status^a^						
Not somked at least 100 cigarettes in life	15.573(2.459,98.631)	0.004	20.083(3.773,106.893)	＜0.001	1.684(0.108,26.278)	0.710

^~^The Pattern II UADCG was the reference

^a^The Male, College graduate or above, Living with partner, Smoked at least 100 cigarettes in life were the reference

## Discussion

This paper takes Hypertension, Dyslipidemia, Diabetes, CKD, and Hyperuricemia as the nodes of KDLG (Pattern IV), combines aging-related biochemical indicators, demographic data, and lifestyle indicators as node features, and constructs a CMM graph network using cosine similarity between nodes. Four CMM patterns are ultimately identified through the Louvain community detection algorithm. These four CMM patterns exhibit significant differences in disease distribution, population characteristics, lifestyles, and nutrient intake. Additionally, this paper compares the performance of six machine learning algorithms in CMM pattern recognition, with Logistic Regression and GradientBoosting algorithms performing exceptionally well. The features playing crucial roles in CMM pattern recognition include Choline intakes, Iron intakes, Niacin intakes, Cholesterol intakes, Vitamin B2 intakes, and Potassium intakes.

Four CMM patterns are isolated in this paper: Hypertension Predominant Group (HPG, Pattern I), Uric Acid and Dyslipidemia Coexistence Group (UADCG, Pattern II), Multiple Diseases High Group (MDHG, Pattern III), and Kidney Disease Low Group (KDLG, Pattern IV). The distribution of these four patterns among the population reveals uneven gender ratios, with a higher proportion of females in MDHG (Pattern III), HPG (Pattern I) and a relatively higher proportion of males in the other patterns. The highest proportion of married individuals is found in HPG (Pattern I), while higher proportions of unmarried or cohabiting individuals are observed in the other patterns. The results of this paper are similar to those found by Drury et al. [32] that older female populations are more susceptible to hypertension, Fang et al., Liu et al., and others that males are more prone to hyperuricemia, and Spohn et al. that males are more susceptible to lipid metabolism abnormalities [33–35]. The possible reason why females are more likely to suffer from the comorbidities of the HPG (Pattern I) pattern is that due to the protective effect of estrogen in the body, females have a relatively low risk of hypertension before menopause. However, after menopause, the decline in estrogen levels weakens its cardiovascular protective effect, reduces its inhibition of the renin–angiotensin–aldosterone system (RAAS), and weakens its role in maintaining vasodilation and endothelial function, increasing the risk of hypertension [36, 37]. In contrast, males are more likely to suffer from UADCG (Pattern II) and KDLG (Pattern IV), which may be related to differences in DNA methylation susceptibility, immune responses (such as interleukin levels), and hormones between males and females, making males more prone to metabolic abnormalities and thus comorbidities related to metabolic abnormalities such as dyslipidemia and hyperuricemia [38]. These gender differences in CMM patterns may also be related to differences in lifestyle and emotional stress between males and females [39]. Furthermore, this paper finds that the age of HPG (Pattern I) is significantly higher than that of other patterns, while the age of KDLG (Pattern IV) is significantly lower than that of MDHG (Pattern III). This is similar to previous findings that CMM patterns with hypertension and CKD usually occur in older age groups, while CMM patterns with dyslipidemia and CKD usually occur in younger age groups [40]. Possible reasons for this phenomenon include the following: Dyslipidemia can lead to the deposition of lipids in islet cells, affecting glucose oxidation, and subsequently triggering β-cell apoptosis and insulin resistance. Additionally, dyslipidemia reduces glucose utilization and increases glycogenolysis in the liver, thereby exacerbating insulin resistance [41, 42]. Insulin resistance then triggers inflammation, oxidative stress, insulin receptor mutations, endoplasmic reticulum stress, and mitochondrial dysfunction. These pathological processes impair endothelial function in arteries, leading to the occurrence of hypertension, etc. [43, 44]. Another explanation is that dyslipidemia can promote the deposition of lipids on the blood vessel wall, triggering inflammation and vascular plaques, narrowing the vessel diameter and restricting blood flow, leading to the occurrence of hypertension, etc. [45, 46].

This paper finds that the proportion of patients in the UADCG (Pattern II) pattern who have smoked over 100 cigarettes is the highest. This is consistent with findings by Gee et al. (2016) [47], Kim et al. (2019) [48], Kim et al. (2013) [49], and Momayyezi et al. (2024) [50] that smoking is linked to increased serum uric acid levels and dyslipidemia. The possible association between smoking and the UADCG (Pattern II) pattern stems from a series of complex pathogenic mechanisms, including smoking promoting the production of reactive oxygen species, which leads to oxidative stress reactions in key molecules such as lipids and proteins, causing cell damage. Meanwhile, smoking activates pro-inflammatory responses, directly threatening health. It also interferes with metabolic pathways by releasing cytokines and inflammatory mediators, affecting uric acid excretion and synthesis, leading to increased uric acid levels and hyperuricemia. At the same time, these inflammatory substances also regulate fat metabolism, disrupting the balance of lipoprotein synthesis and decomposition, and subsequently causing abnormal blood lipid levels [51, 52].

This paper finds that there are significant differences in nutrient intake among patients with different CMM patterns, and among the top six features affecting CMM pattern recognition, all are nutrient intake levels, specifically including Choline intake, Iron intake, Niacin intake, Cholesterol intake, Vitamin B2 intake, and Potassium intake. Elevated choline intake is associated with an increased risk of comorbidity in HPG (Pattern I) and KDLG (Pattern IV) patterns. Increased iron intake is linked to a higher risk of comorbidity in the KDLG (Pattern IV) pattern. Conversely, higher cholesterol intake is correlated with an elevated risk of comorbidity in the HPG (Pattern I) pattern. Mechanistically, choline, as a structural component of cell membranes and a precursor to the neurotransmitter acetylcholine, influences lipid metabolism and membrane stability, thereby modulating the pathogenesis of conditions such as hypertension and chronic kidney disease [53, 54]. Iron, serving as a core constituent of hemoglobin and myoglobin, exhibits metabolic dysregulation that functions as an inflammation marker associated with chronic diseases, and chronic inflammatory states demonstrate a potential inverse relationship with the risk of renal diseases [55]. Niacin, necessary for energy metabolism, influences blood lipids, uric acid, and renal function [56]. Cholesterol, crucial for cellular integrity and hormone balance, impacts inflammation, blood lipids, renal function, and type 2 diabetes [57, 58]. Vitamin B2 participates in energy metabolism and antioxidant defense [59]. Potassium, essential for electrolyte balance and neuromuscular excitability, affects blood pressure, heart function, and is linked to arterial calcification and renal function [60]. It is due to the different physiological mechanisms of these nutrients that they play crucial roles in distinguishing different CMM patterns.

To describe the population distribution characteristics of CMM patterns, this paper uses the NHANES public dataset. Due to dataset limitations, the types of cardiometabolic diseases included in this paper are limited, which to some extent constrains the division of CMM patterns. Additionally, this paper cannot achieve causal inference.

## Conclusions

This paper establishes a recognition framework for Cardiovascular and Metabolic Diseases (CMM) patterns that integrates demographic data, lifestyle indicators, and biochemical indicators. Initially, we constructed a CMM graph network dataset and applied the Louvain algorithm for community detection, thereby identifying and classifying four CMM patterns: Hypertension Predominant Group (HPG, Pattern I), Uric Acid and Dyslipidemia Coexistence Group (UADCG, Pattern II), Multiple Diseases High Group (MDHG, Pattern III), and Kidney Disease Low Group (KDLG, Pattern IV). Based on this, we conducted an in-depth analysis of the distribution characteristics of these CMM patterns across different populations, meticulously comparing the differential impacts of factors such as gender, age, socioeconomic status, and lifestyle on them. Subsequently, this paper compares and evaluates the performance of six machine learning algorithms in CMM pattern recognition tasks and delves into the key features that influence the recognition results. The findings reveal that the intake of nutrients such as choline, iron, niacin, cholesterol, vitamin B2, and potassium plays a crucial role in the process of CMM pattern recognition. Future research can incorporate more cardiovascular and metabolic diseases and further analyze the mechanisms by which nutrient intake influences CMM patterns, providing a more solid theoretical and practical foundation for personalized health management. Future research can incorporate more cardiovascular and metabolic diseases and further analyze the mechanisms by which nutrient intake influences CMM patterns, providing a more solid theoretical and practical foundation for personalized health management.

## Data Availability

The data was avaliable at https://www.cdc.gov/nchs/nhanes/.
